# A Proliferating Trichilemmal Tumor at an Uncommon Site Treated With Radical Excision: A Case Report and Literature Review

**DOI:** 10.7759/cureus.64803

**Published:** 2024-07-18

**Authors:** Ahmad N Boeisa, Abdullah M Alkhars, Alreem A Albaqshi, Mohammed S Al-Arbash, Mahfood A Alqatari, Issa A Mohammad Mousa

**Affiliations:** 1 Orthopedic Surgery, Almoosa Specialist Hospital, Al-Ahsa, SAU; 2 Orthopedic Surgery, King Fahad Hospital Hofuf, Al-Ahsa, SAU; 3 College of Medicine, King Faisal University, Al-Ahsa, SAU; 4 Histopathology, Almoosa Specialist Hospital, Al-Ahsa, SAU; 5 Anaesthesia, Almoosa Specialist Hospital, Al-Ahsa, SAU

**Keywords:** pilar tumor treatment, clinical features of proliferating trichilemmal tumor, therapeutic approach of proliferating trichilemmal tumor, benign proliferating trichilemmal tumor, pathology features of pilar tumor, pathology features of proliferating trichilemmal tumor, rare pilar tumor site, pilar cyst, pilar tumor, proliferating trichilemmal tumor

## Abstract

A proliferating trichilemmal tumor (PTT) is a rare, benign, exophytic tumor originating from the isthmus region of the outer root sheath of the hair follicle. Clinically, PTTs manifest as isolated, exophytic, firm nodules that have the potential to ulcerate. These tumors may occasionally originate from a pre-existing trichilemmal cyst, or they can emerge spontaneously. Most exclusively these lesions are seen on the scalp. However, rarely these tumors can be found in other anatomical areas. Our patient had a protruding mass in her shoulder for 20 years, and this is a rare site for the occurrence of these lesions; it could be the first case to document such a site, as far as we found in the literature. The mainstay treatment of the PTT is surgical excision of the tumor, assessing the histological margins to ensure sufficient resection was made, close monitoring, and follow-up with the patient.

## Introduction

A proliferating trichilemmal tumor (PTT) is a rare, benign, exophytic tumor originating from the isthmus region of the outer root sheath of the hair follicle [1,2]. The PTT was initially described in a clinical context by Wilson Jones in 1966 and was originally named the "proliferating epidermoid cyst" [3]. The PTT has been identified and reported in the literature under various names, such as proliferating pilar tumor, giant hair matrix tumor, invasive hair matrix tumor of the scalp, invading pilomatrixoma, trichilemmal pilar tumor, trichochlamydoacanthoma, and squamous cell carcinoma originating from sebaceous cysts [4,5]. Clinically, PTTs manifest as isolated, exophytic, firm nodules that have the potential to ulcerate [6,7]. These tumors may occasionally originate from a preexisting trichilemmal cyst, or they can emerge spontaneously [2,8]. Microscopically, it is characterized by a partly cystic and solid lesion featuring enlarged keratinocytes, sudden keratinization, a lack of a granular layer, and varying degrees of cytologic atypia [7,8]. 

About 90% of reported PTT cases are solitary lesions on the scalp [5,9]. However, these tumors can be found in other anatomical areas, such as the face, ear, neck, shoulder, upper extremities, trunk, anogenital area, buttocks, and lower extremities [4-6,9-12]. The size of PTT lesions typically ranges from less than 1 cm to 10 cm in diameter but can increase if left untreated [11]. The largest tumor size reported in the literature was 25 cm in diameter [13]. Furthermore, the majority of reported cases of these tumors are seen in women over the age of 60, with a 2.5:1 female-to-male ratio [6].

The diagnosis of PTTs mainly relies on their histological features of lobulated clusters of squamous cells forming both solid and cystic regions, often connected to the epidermis [6,7]. Trichilemmal keratinization is another characteristic feature of PTTs, which is the abrupt transition from nucleated epithelial cells to anucleated cells without a granular layer [3,5,9]. Moreover, these tumors may mimic squamous cell carcinoma, making histological differentiation challenging [1,14]. Despite this, PTTs generally have a benign clinical course, with few studies reporting aggressive behavior with local recurrences and metastasis [4,10,15,16].

The most common therapeutic approach for PTTs includes surgical excision of the tumors, evaluating the histological margins to confirm adequate resection, and maintaining careful, close follow-ups [6,17]. Radiation and chemotherapy have been proposed as alternative treatments; however, medical data and reports are limited regarding this in the literature [16-21]. Although some studies reported wide local excision as the treatment of choice for PTTs, no set standards were found in the literature regarding the recommended margins and depth of the excision in case of malignant tumors [4,21,22]. However, a surgical excision with a 1 cm margin of healthy tissue is sufficient.

## Case presentation

An 82-year-old woman complained of a protruding mass in her right shoulder for 20 years and came to our outpatient orthopedic clinic after seeing several other physicians and hospitals. Over the past six months, the swelling has worsened, grown larger, and is painful. She claimed that some cheesy material exudated when she squeezed. She had no prior medical history of cysts, nor did her family history. Neither fever nor weight loss was associated with it. However, the pain, disfiguring, and mass size had an impact on her shoulder range of motion, quality of life, ability to sleep, and mood.

Due to chronic cough complaints, the patient was referred to a pulmonologist for additional evaluation and preoperative recommendation. The pulmonary clinic assessment showed no active respiratory symptoms, 96% oxygen saturation, and a clear chest examination. However, she was cleared to proceed with the mass excisional biopsy of her right shoulder as well as additional care and monitoring for the lung nodule, which was incidentally discovered and will be biopsied using CT guidance under general anesthesia.

Lesion description and examination

**Figure 1:**
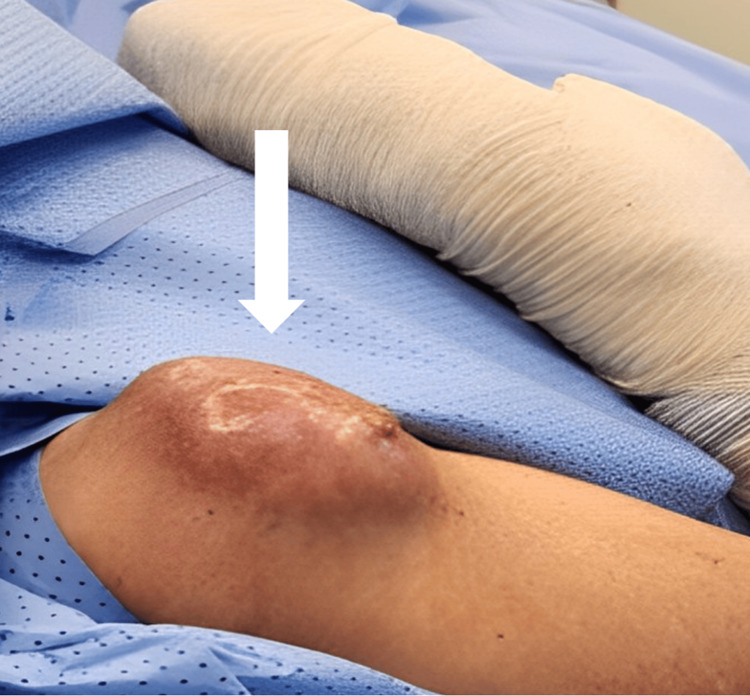
Pre-OP tumor appearance The white arrow shows a well-defined sizable mass lesion. It is a hard, freely mobile, tender mass with hotness and redness

**Figure 2:**
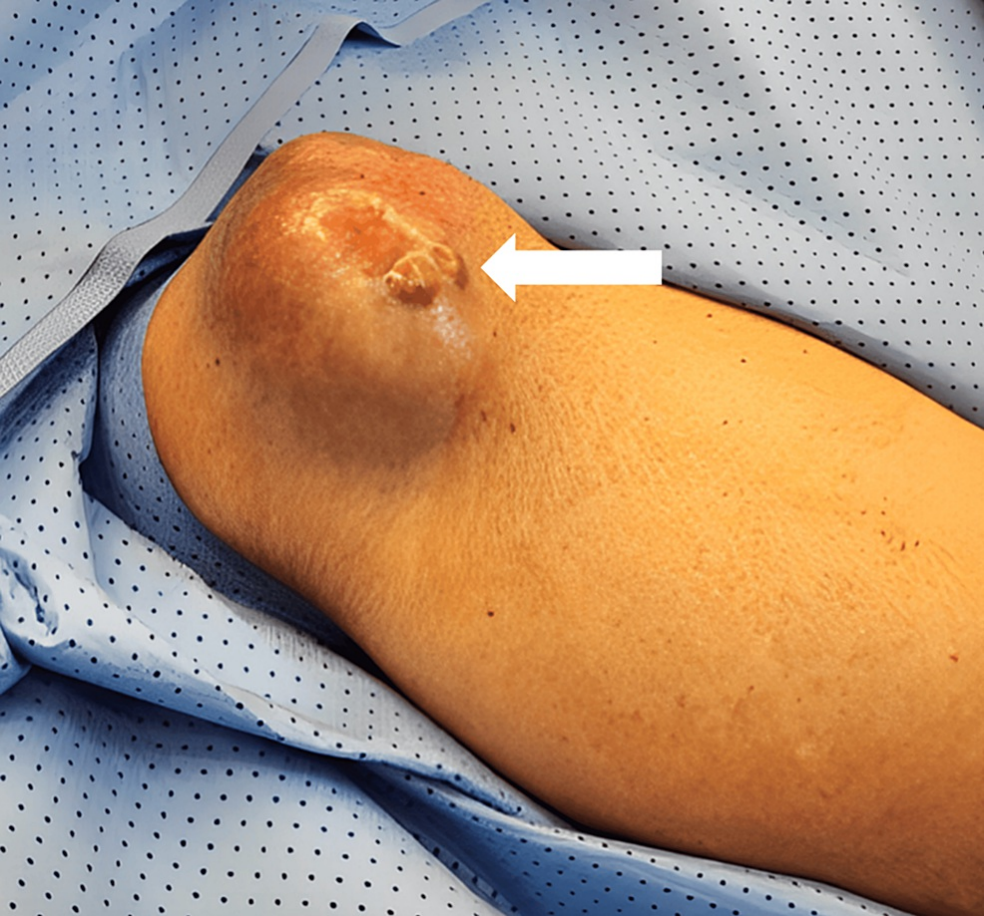
Additional nodules The white arrow shows a sizable mass lesion, and the tumor exhibited two additional exophytic nodules and measured approximately 0.5 x 0.5 cm each

On examination, a well-circumscribed, violaceous, exophytic tumor measuring 5.0 x 6.0 cm at its base was identified on the right surface of the shoulder. The tumor exhibited two additional, smaller exophytic nodules on its surface. These nodules were soft, mobile, and approximately 0.5 x 0.5 cm each, with minimal purulent discharge (Figures 1, 2). Palpation revealed a hard, freely mobile, tender mass with hotness and redness. There was no lymphadenopathy.

Investigations

**Figure 3:**
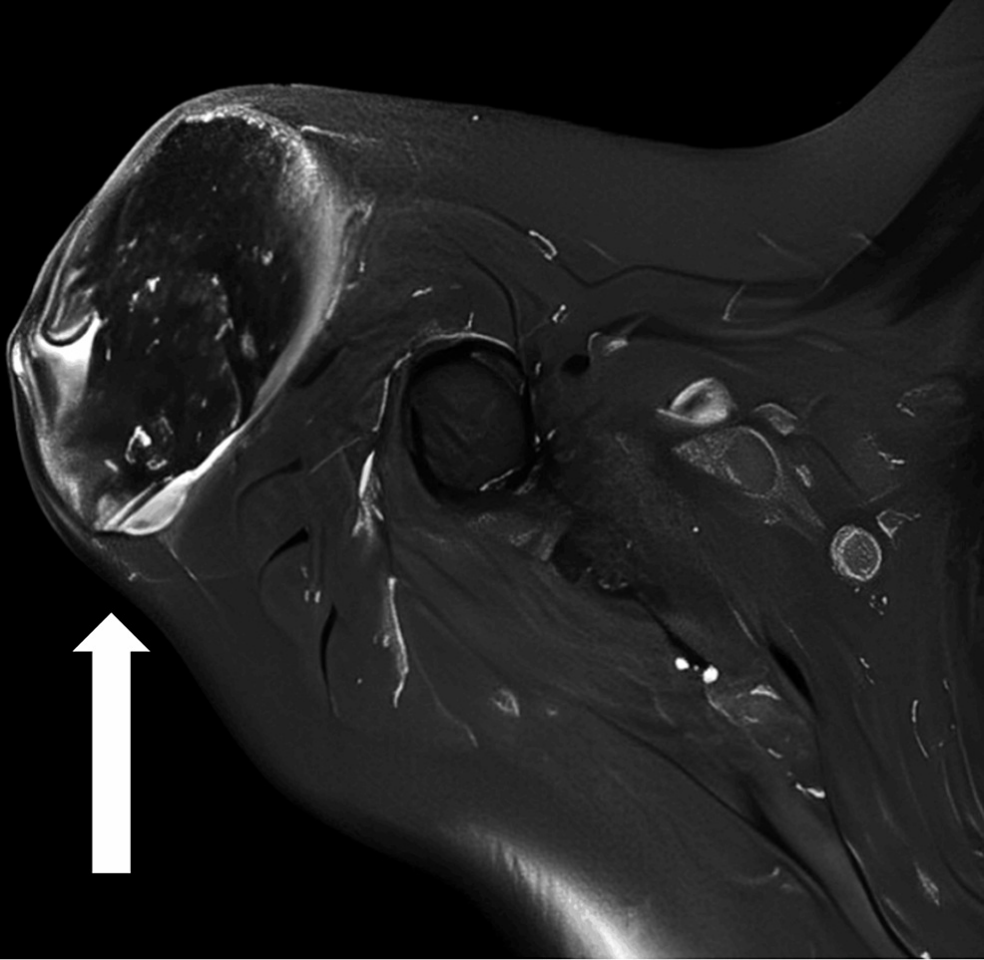
MRI axial view The white arrow shows a well-defined sizable mass lesion

**Figure 4:**
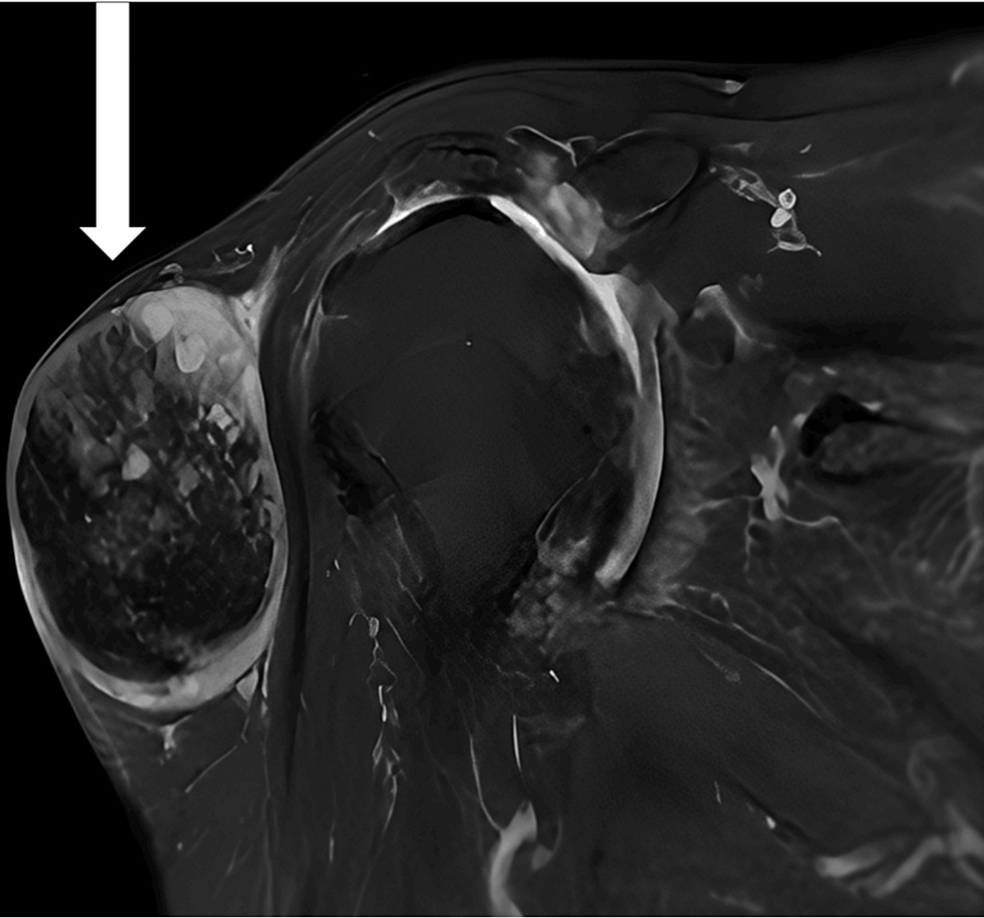
MRI coronal view The white arrow shows a well-defined sizable mass lesion with hemorrhagic/calcific foci compressing the lateral aspect of the deltoid muscle

**Figure 5:**
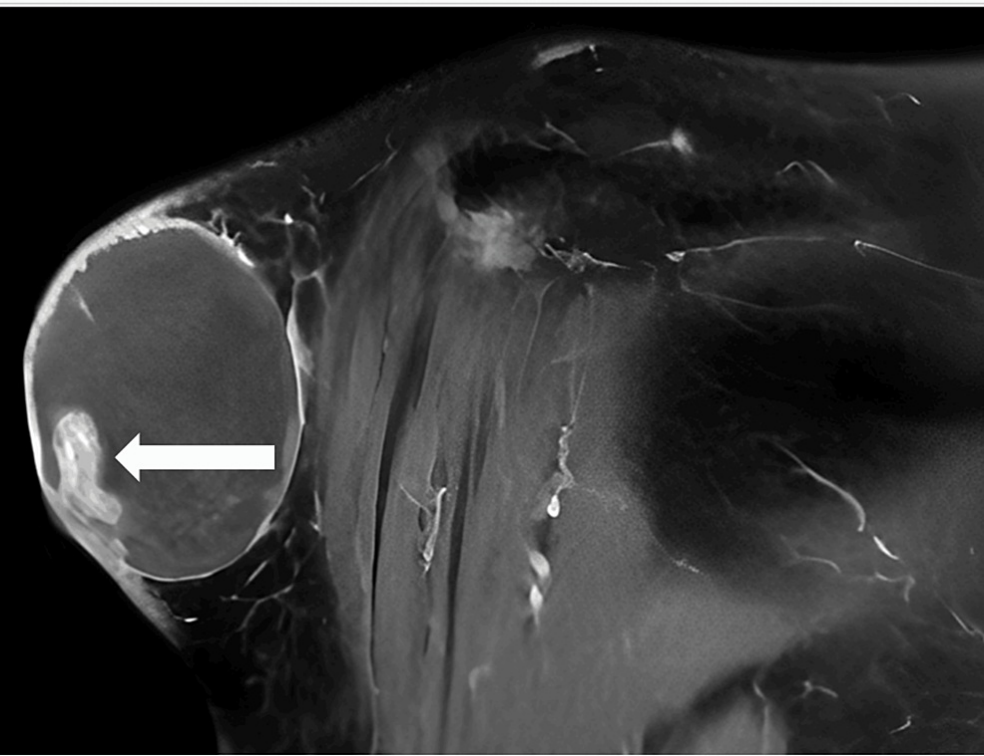
MRI showed compressing on the lateral aspect of the deltoid muscle The white arrow shows evidence of hemorrhagic/calcific foci

MRI with contrast under sedation showed evidence of a right shoulder SC well-defined sizable mass lesion (5.6 x 3.8 x 4.7 cm) with hemorrhagic/calcific foci compressing the lateral aspect of the deltoid muscle with no evidence of invasion to the surrounding structure (Figures 3-5).

**Figure 6:**
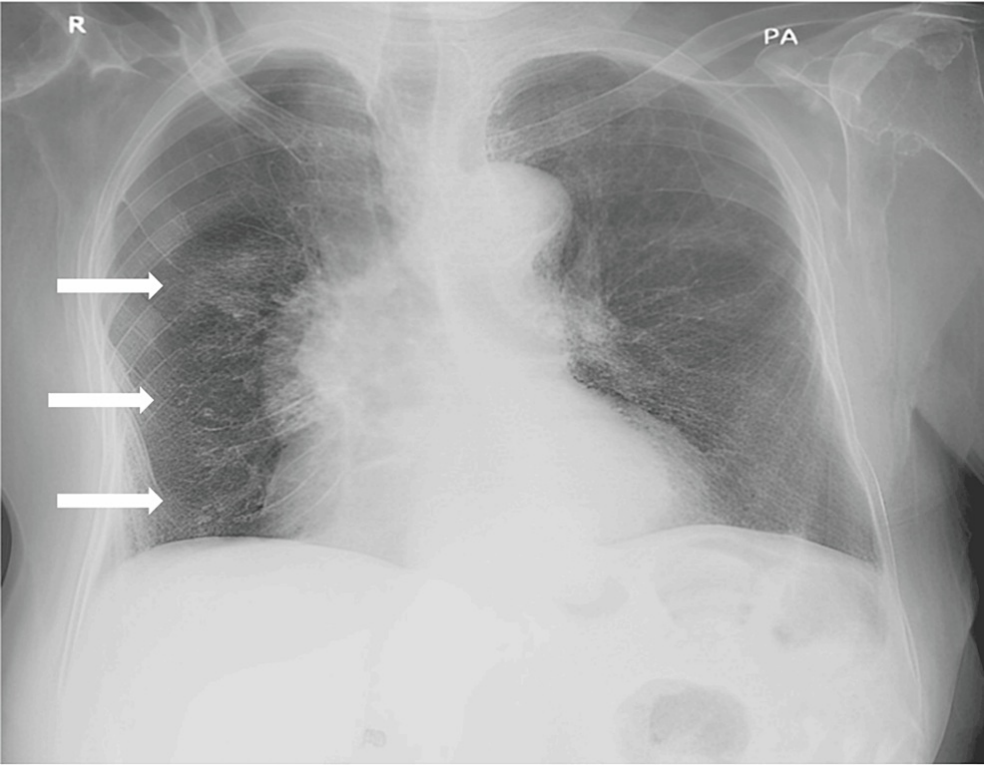
PA chest X-ray The white arrows shows scattered small, calcified granulomas in the right lung with fibrotic bands

**Figure 7:**
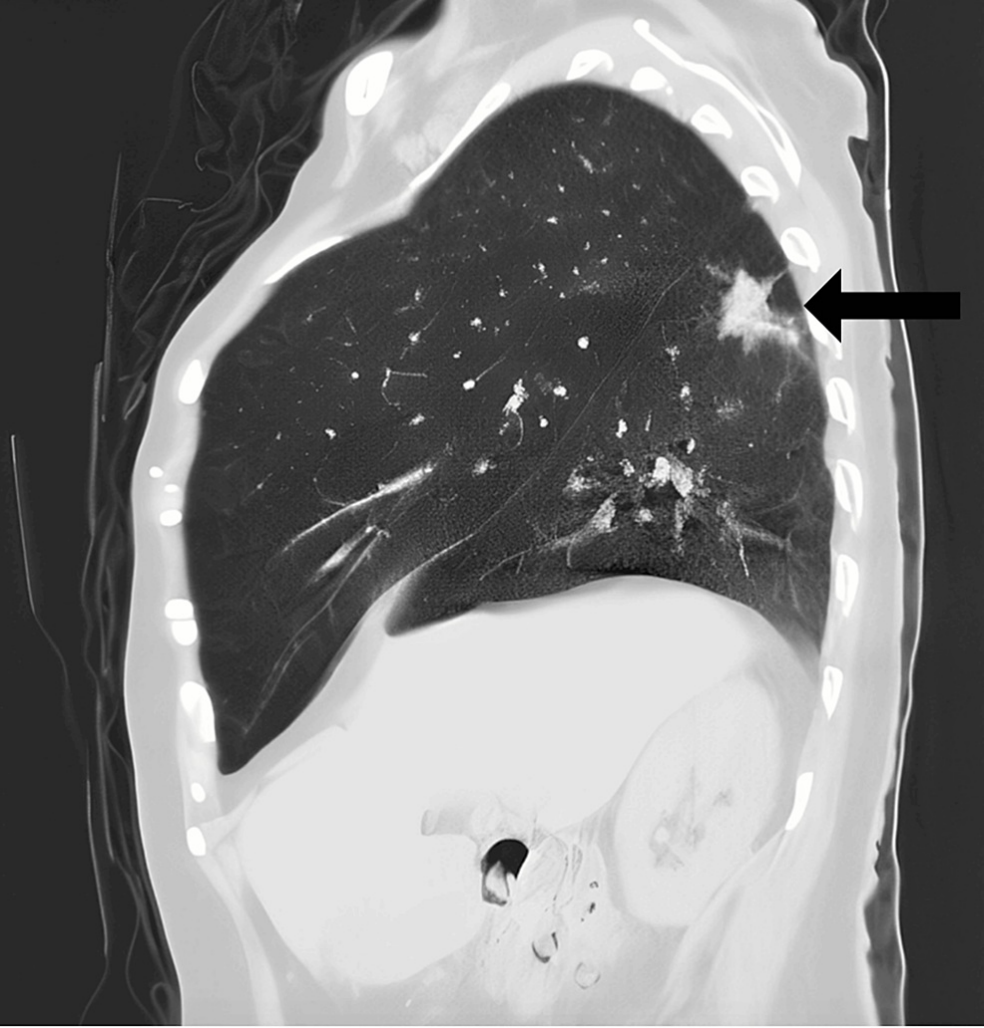
Chest CT sagittal view The black arrow shows a posterior superior nodule. In addition, there are scattered small, calcified granulomas in the right lung with fibrotic bands at the bases

**Figure 8:**
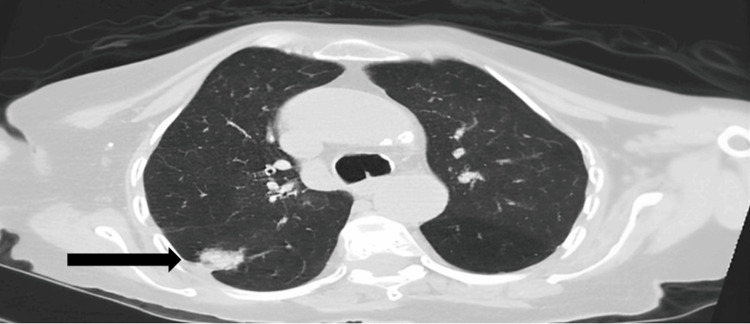
Axial chest CT The black arrow shows a right upper lung posterior lobulated, spiculated nodule

**Figure 9:**
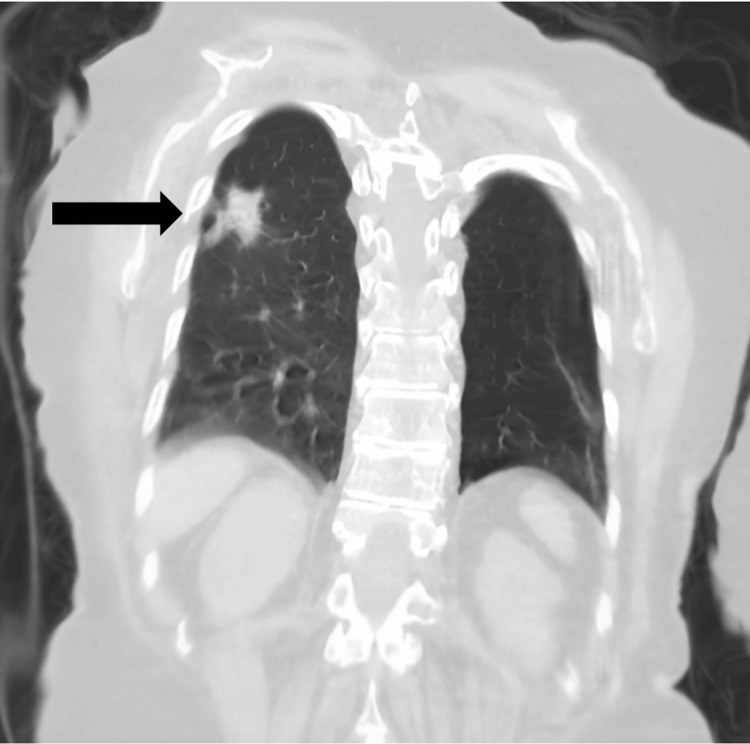
Chest CT coronal view The black arrow shows a right lung lobulated, spiculated nodule measuring around 2.5×2 cm

A radiologic chest X-ray exam, and CT scan without contrast showed a lobulated, spiculated nodule in the superior segment of the right lower lobe measuring around 2.5×2 cm. In addition, there were scattered small, calcified granulomas in the right lung with fibrotic bands at the bases (Figures 6-9).

Management

The patient was sedated with 20 mg ketamine and 1 mg midazolam. Then, right ultrasound-guided Interscaline block with 15 ml bupivacaine 0.5% was done. In the OR, the patient was on propofol infusion 45 mcg/kg/min all over the procedure.

**Figure 10:**
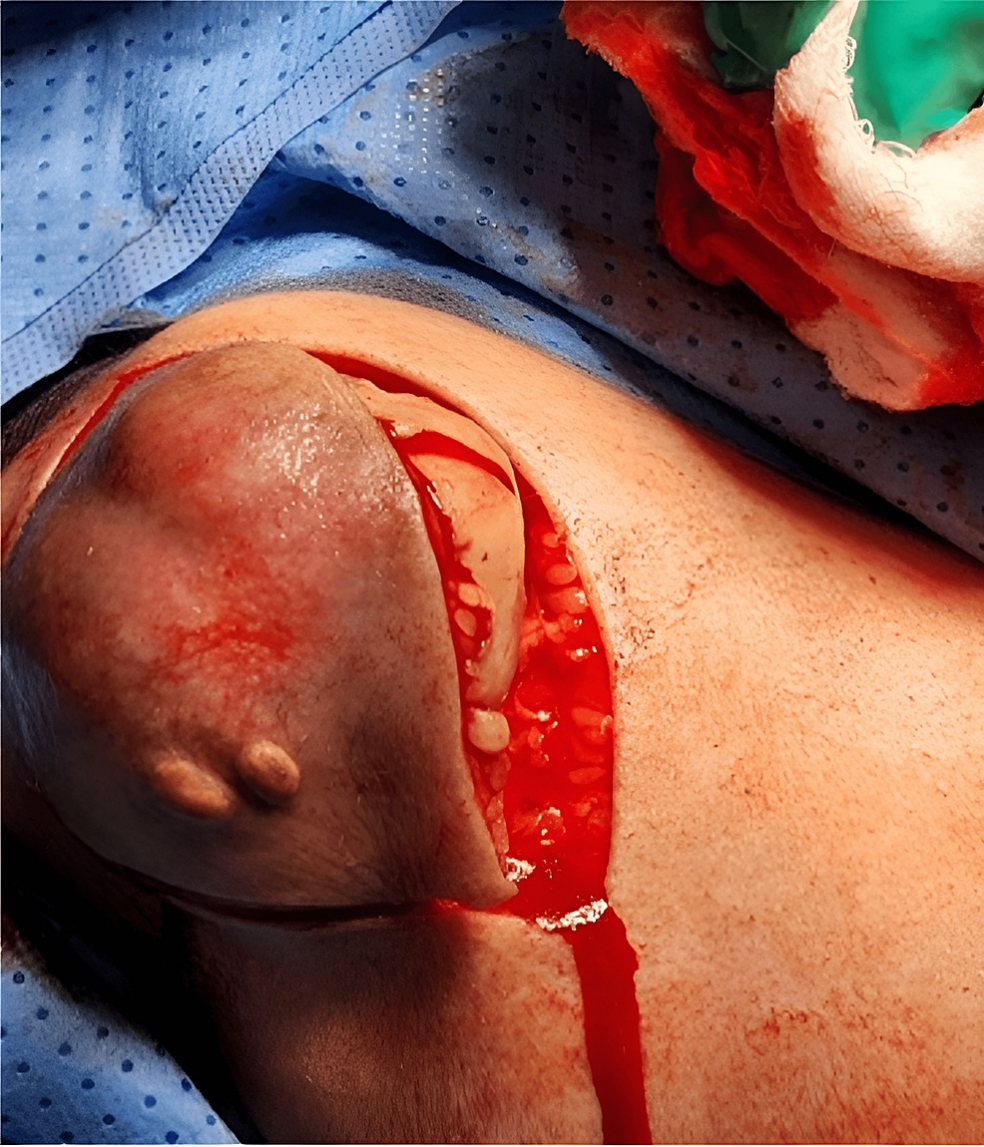
An elliptical incision

**Figure 11:**
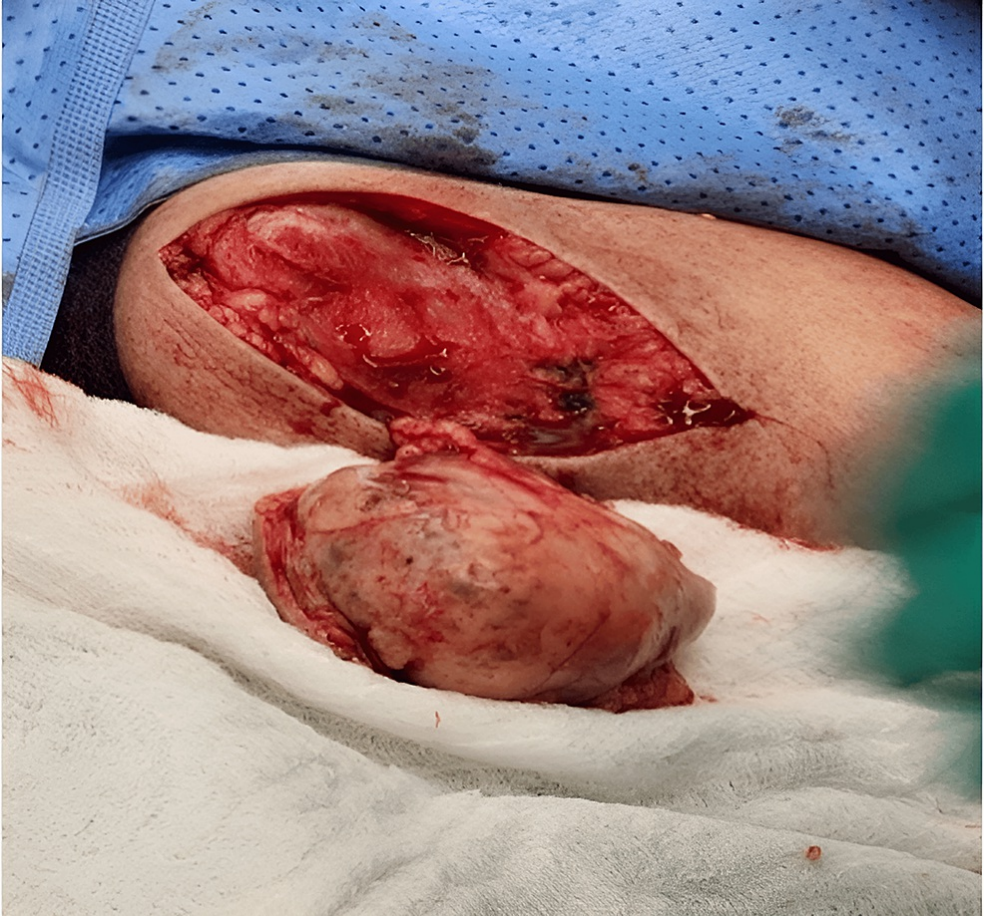
En bloc resection was made

**Figure 12:**
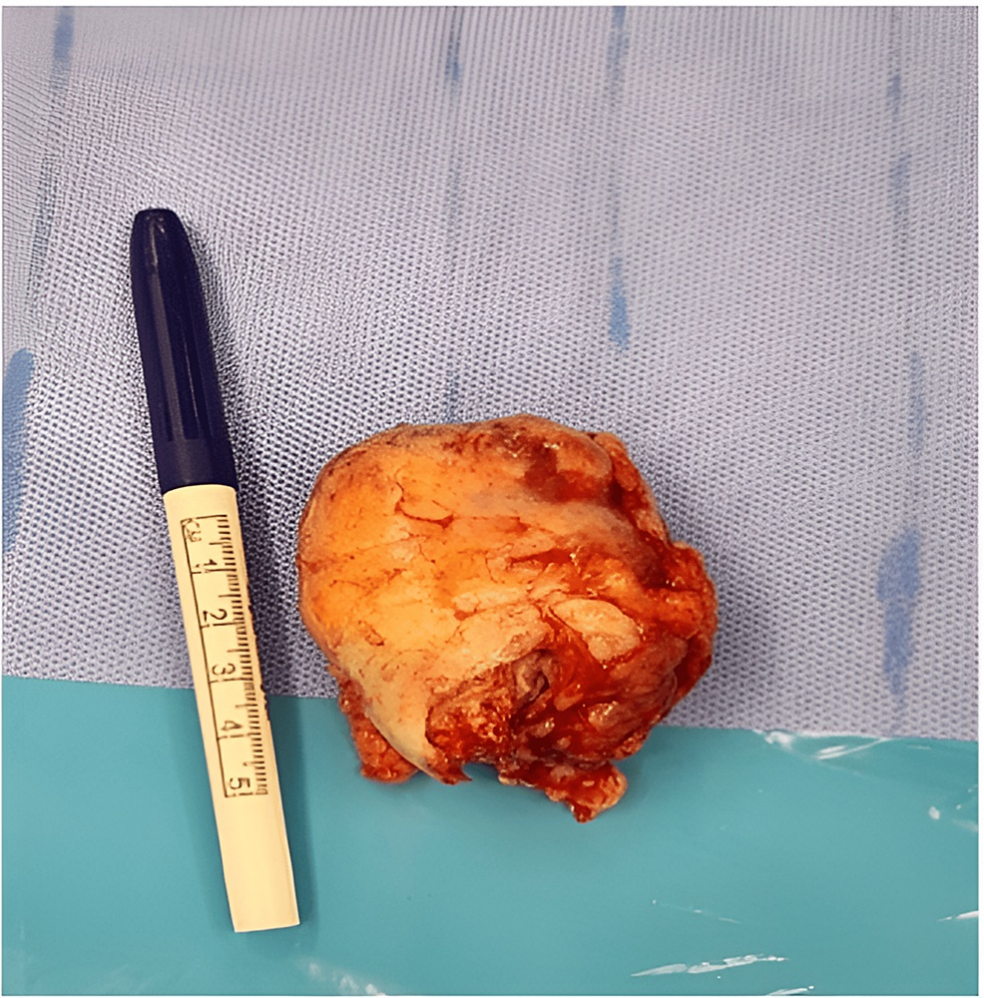
Tumor gross appearance without violation of its capsule

**Figure 13:**
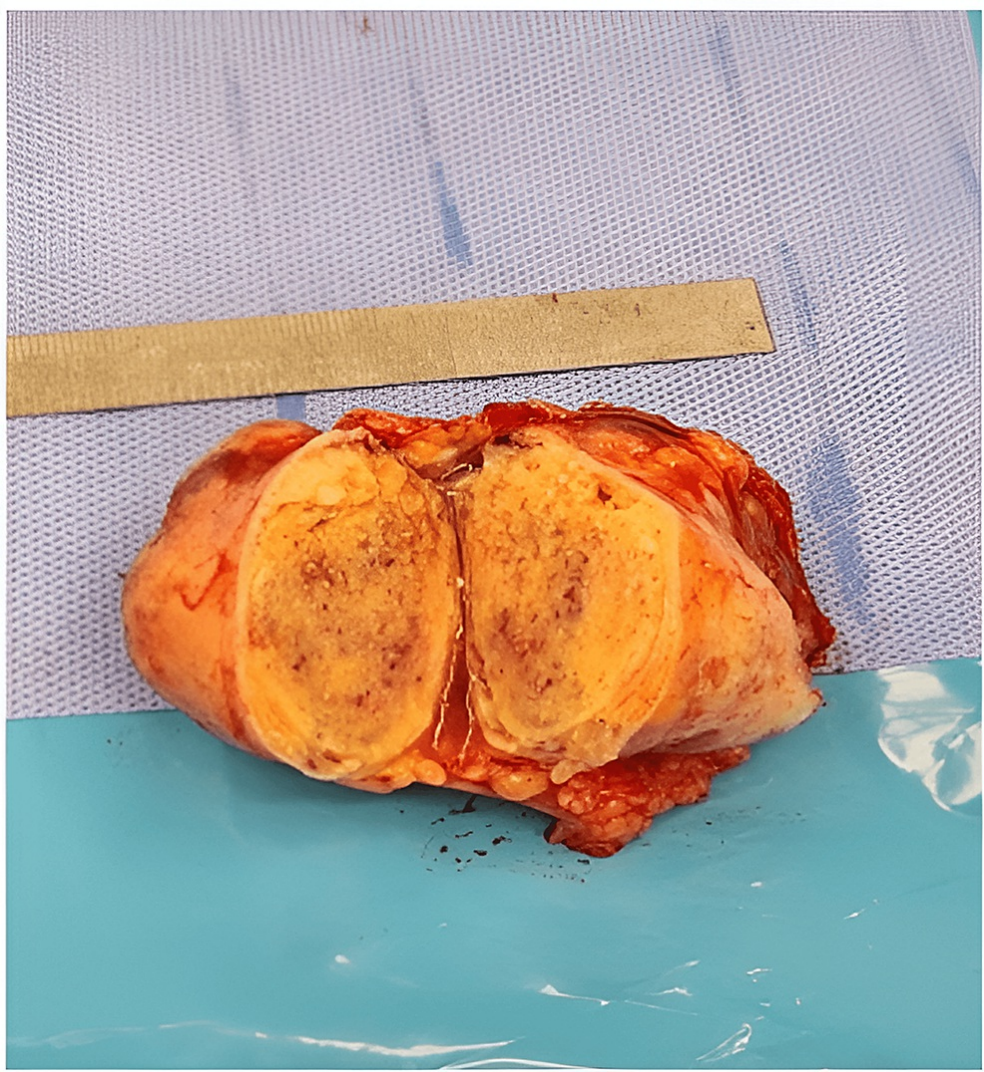
Encapsulated mass with a smooth tan-white external surface measuring 6 x 5 x 3 cm

She was in a supine position and her shoulder was elevated. An elliptical incision was made (Figure 10), and radical excision of the tumor and en bloc resection (Figure 11), without violation of its capsule (Figure 12), were made. The specimen was then sent for histopathology (Figure 13).

The patient was discharged a few hours later as the operation was done as a day case. 

Histopathology

**Figure 14:**
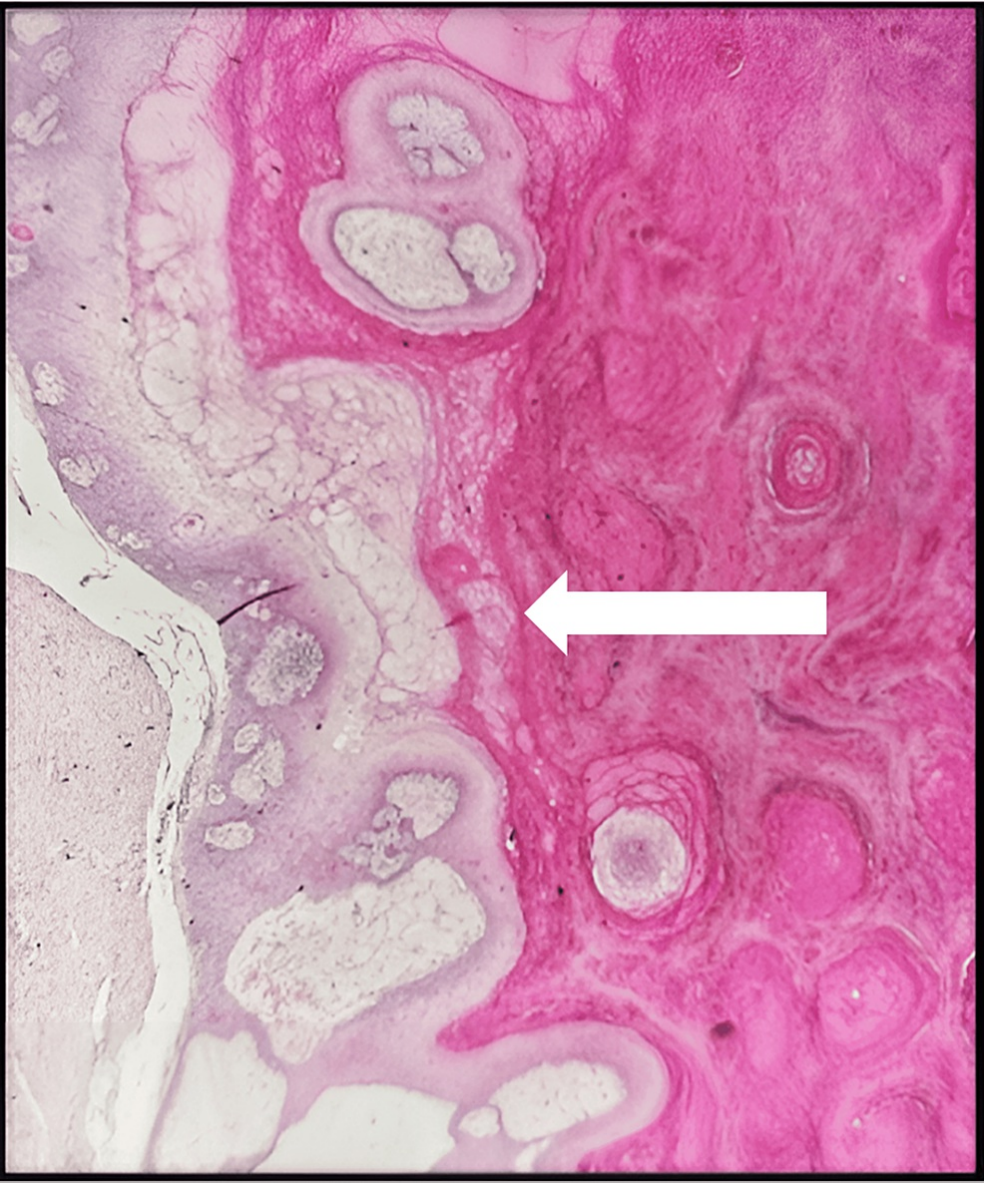
First histopathology section The white arrow shows a well-circumscribed, large, multicystic, dermal-based tumor

**Figure 15:**
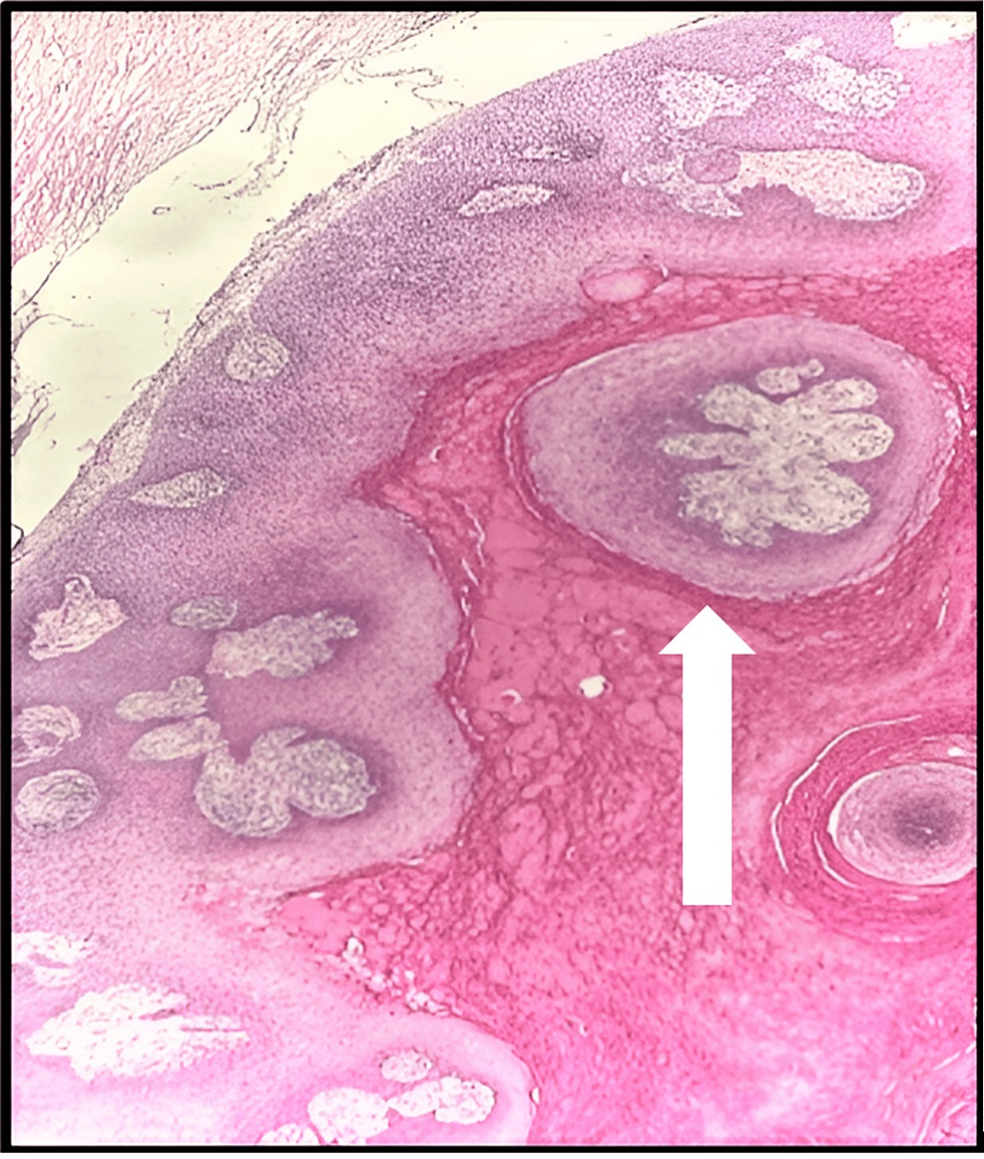
Microscopic examination of the second section The white arrow shows a dermal-based tumor composed of lobules of proliferative squamous epithelium showing trichilemmal keratinization

**Figure 16:**
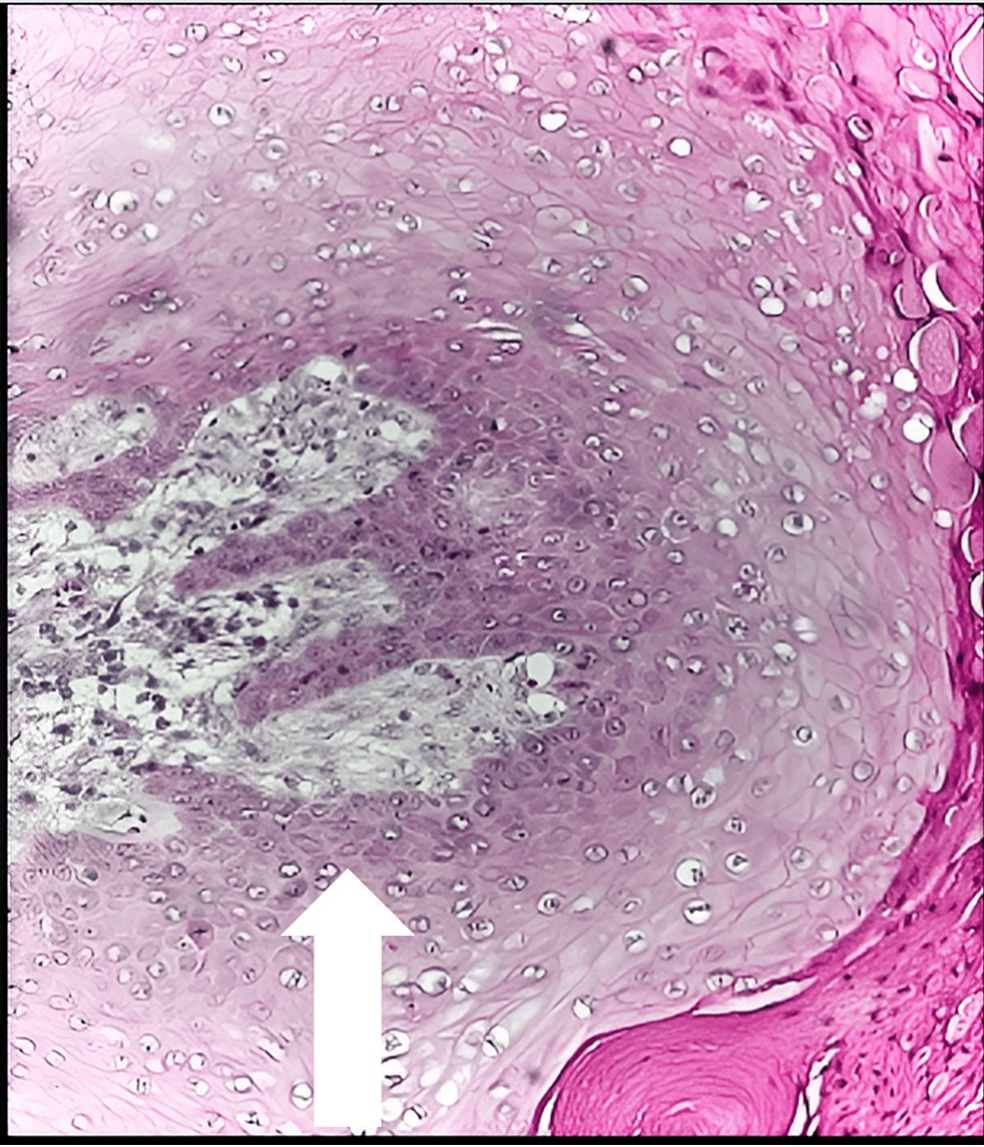
Microscopic examination of the histologic section The white arrow shows that the squamous cells demonstrate abundant eosinophilic cytoplasm and mildly enlarged and hyperchromatic staining nuclei. No high-grade atypia or increased mitotic activity is seen.

Low- and high-power images of H&E stain show large, cystic, dermal-based tumor composed of lobules of proliferative squamous epithelium. The squamous cells demonstrate abundant eosinophilic cytoplasm and mildly enlarged nuclei. The epithelium shows trichilemmal keratinization with an absent granular layer. No high-grade atypia or increased mitotic activity was seen (Figures 14-16).

Follow-up

**Figure 17:**
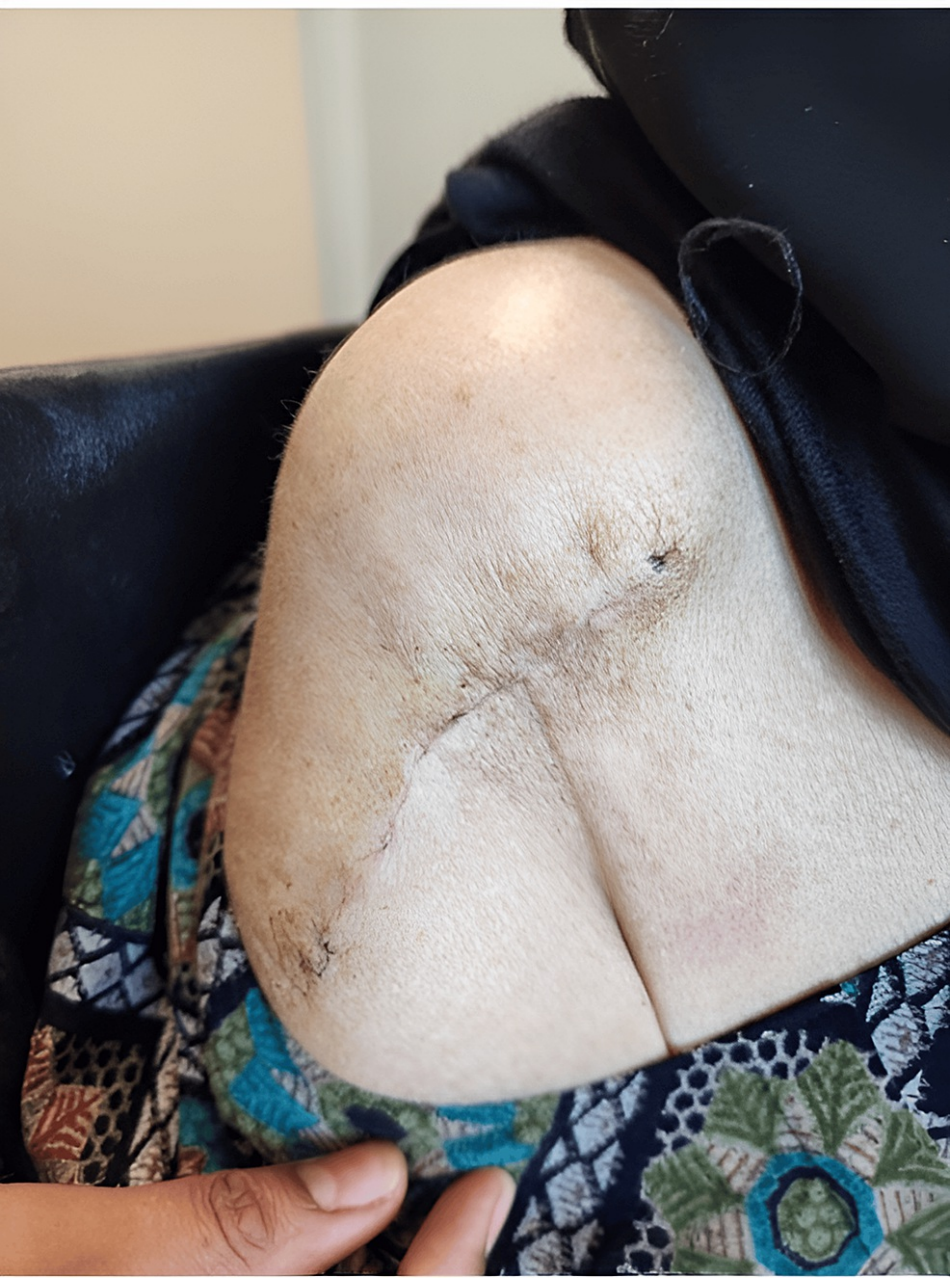
Three weeks later, the surgical wound looked healthy

Three weeks after the surgery, the patient visited the clinic. She was enthusiastic and happy, and her surgical wound had healed and was free of redness and discharge (Figure 17). She also had a full range of motion in her shoulder and the distal neurovascular was intact.

## Discussion

Less than 100 cases of the PTT, a rare neoplasm, have been reported in the 50 years since it was first identified [23]. It was previously believed to be a type of squamous cell carcinoma (SCC) originating from a sebaceous cyst. Histologically, it may resemble SCC. The absence of a premalignant epidermal lesion and a granular layer of cells are the characteristic traits that favor the PTT over SCC [1-5]. Our case pathologic examination is consistent with the literature on PTT features: well-circumscribed, large, multicystic, dermal-based cell, composed of lobules of proliferative squamous epithelium, showing trichilemmal keratinization. The squamous cells may demonstrate abundant eosinophilic cytoplasm with enlarged hyperchromatic staining nuclei, with no high-grade atypia or increased mitotic activity should be seen. The lesion in our case had well-defined borders and did not infiltrate the surrounding tissue. However, the nature of these lesions can also show wide areas of rapidly expanding epithelial cells. Moreover, large, long-lasting, or rapidly progressing lesions may result in fungal infection of the epithelium above. In the last stage, total loss of p53 has been identified as one mechanism through which a PTT can develop into a malignant tumor [24]. Subsequently, there is severe cellular atypia and invasion into the surrounding tissue. However, a CT scan with contrast, a chest X-ray, and a whole-body PET scan are recommended as possible work-ups if metastatic disease is suspected. These tests help rule out distant metastatic disease [24]. 

About 90% of PTT cases that have been reported involve solitary scalp lesions [5-9]. On the other hand, our patient had a protruding mass in her shoulder, which is a rare site for occurrence of these lesions, and this is the first case to document such a site, as far as we found in the literature. However, other findings, which include age, gender, and size of the tumor, are in agreement with previous reports. Lesions from the PTT usually have a diameter of less than 1 cm to 10 cm. The largest tumor size ever documented in the literature had a diameter of 25 cm. Moreover, women over 60 account for the majority of documented cases [4-6,9-12].

The mainstay treatment of PTTs is surgical excision of the tumor, assessing the histological margins to ensure sufficient resection was made, and closely monitoring and following the patient up [6,17]. Other treatment options like radiation and chemotherapy have been suggested, but there is a lack of scientific medical data and information on this in the literature [16-24]. However, the essential regimen in case of malignant PTTs that have not spread is surgical excision with a 1 cm safety margin [16-24]. Additionally, some authors advise considering neoadjuvant radiation therapy in case of advanced age with the aim to minimize the size of the tumor, followed by surgical resection, especially if it is in a region that is sensitive functionally or cosmetically, and they achieved an inspiring outcome [24].

## Conclusions

PTT lesions can also occur in other anatomical locations, including the face, ear, neck, shoulder, upper limbs, trunk, anogenital area, buttocks, and lower extremities, even though most of them are isolated lesions on the scalp. Typically, the lesion had clearly defined boundaries and did not infiltrate the adjacent tissue. Although they are benign, if metastatic disease is suspected, a whole-body PET scan, a chest X-ray, and a CT scan with contrast are advised. Surgical resection, evaluation of the histological margins to confirm adequate resection, thorough patient monitoring, and follow-up are the cornerstones of PTT treatment. Since there is a lack of scientific medical data on the effectiveness of various treatment methods, such as radiation and chemotherapy, in the literature, further research is required.
